# Biallelic mutations of *EGFR* in a compound heterozygous state cause ectodermal dysplasia with severe skin defects and gastrointestinal dysfunction

**DOI:** 10.1038/s41439-018-0011-0

**Published:** 2018-06-08

**Authors:** Shion Hayashi, Takayuki Yokoi, Chihiro Hatano, Yumi Enomoto, Yoshinori Tsurusaki, Takuya Naruto, Masahisa Kobayashi, Hiroyuki Ida, Kenji Kurosawa

**Affiliations:** 1 Department of Pediatrics, The Jikei University School of Medicine, Tokyo, Japan; 2 Division of Medical Genetics, Kanagawa Children’s Medical Center, Yokohama, Japan; 3 Clinical Research Institute, Kanagawa Children’s Medical Center, Yokohama, Japan; 4 Pediatrics and Developmental Biology, Tokyo Medical and Dental University Graduate School, Tokyo, Japan

## Abstract

Epidermal growth factor receptor (EGFR), a receptor that recognizes epidermal growth factor, is a very important regulator of cell proliferation and differentiation. To date, three cases of severe ectodermal dysplasia were reported to be caused by an inherited germline homozygous loss-of-function missense mutation of *EGFR*. This is the first report of a patient with biallelic compound heterozygous mutations in *EGFR*.

Ectodermal dysplasias (EDs) are congenital disorders characterized by the alteration of two or more ectodermal structures. They are phenotypically and genetically heterogeneous^1^. Epidermal growth factor receptor (EGFR), a receptor that recognizes EGF, is a very important regulator of cell proliferation and differentiation^2,3^. Campbell et al.^4^ and Ganetzky et al.^5^ reported on patients with ED who possessed homozygous loss-of-function (LoF) mutations of *EGFR*. These patients had common biallelic homozygous mutations. Here, we report on the first patient who showed a severe ED phenotype and who was found to possess biallelic compound heterozygous mutations in *EGFR*.

The proband was a male infant born at 32 weeks of gestation to healthy, non-consanguineous Japanese parents with no family history of any disease. His APGAR scores were six at 1 min and 8 at 5 min. He weighed 1089 g at birth, with a height of 36 cm and a head circumference of 29 cm. He was intubated and required mechanical ventilation because of respiratory distress on his first day. His respiratory condition was improved and he was removed from mechanical ventilation the next day. At birth, he showed scalp hair defects, a ventricular septal defect, and a right inguinal hernia. He developed recurrent skin disorders such as eczema, anhidrosis, and hyperalgesia after birth. His body temperature was easily affected by environmental temperatures and sometimes elevated over 38 °C in the incubator. His unstable body temperature suggested sweat gland defects. He also presented with diarrhea and electrolyte imbalance (hyposodium and hyperpotassium) beginning on day 40. He showed profound failure to thrive and had tube feeding. However, his body weight only increased 7 g/day, likely because of the diarrhea and electrolyte imbalance, which were suggestive of malabsorption. Hematologic and biochemical findings frequently showed inflammation (white blood cells and/or CRP significantly elevated) during the course without any apparent symptoms, suggesting recurrent bacterial infection. He recovered from each infection following the administration of antibiotics. He died from septic shock resulting from incarceration of the right inguinal hernia on day 134. The patient underwent a postmortem pathological autopsy with the consent of his parents.

Written informed consent was obtained from the parents of the patient in accordance with the guidelines provided by the Kanagawa Children’s Medical Center Review Board and Ethics Committee. Total genomic DNA was obtained from lymphocytes using a QIAamp DNA Blood Mini Kit (Qiagen, CA, USA) following the manufacturer’s instructions. DNA libraries were enriched for sequences using a TruSight One Sequencing Panel (Illumina Inc., San Diego, CA, USA), facilitating enrichment and final analysis of a panel of 4813 genes. The samples were sequenced using a MiSeq sequencing system (Illumina Inc., San Diego, CA, USA), which produced 150-bp paired-end reads. The data were analyzed using the Burrows–Wheeler alignment tool and the Genome Analysis Toolkit pipeline (Broad Institute, Cambridge, MA, USA) and were visualized in the Integrative Genomics Viewer (IGV). Calling of the copy-number variation (CNV) was based on a log–ratio analysis and the *z*-score of read depth for each exon. The variants were confirmed to not be in the human genetic variation database (Japanese genetic variation consortium, a reference database of genetic variations in the Japanese population: 1208 individuals, http://www.genome.med.kyoto-u.ac.jp/SnpDB), the 1000 genomes project, the NHLBI grant opportunity Exome Sequencing Project (ESP) or the Exome Aggregation Consortium (ExAC), and our 600 in-house control Japanese genomic samples. An in silico analysis was performed using ANNOVAR (mainly SIFT (http://sift.jcvi.org/), Polyphen-2 (http://genetics.bwh.harvard.edu/pph2/), and MutationTaster (http://neurocore.charite.de/MutationTaster/)). Mutations identified by targeted sequencing were confirmed by Sanger sequencing and demonstrated appropriate segregation with phenotype in the unaffected parents.

Targeted sequencing identified two novel *EGFR* (NM_005228) mutations: a c.292C > T/p.R98X in exon 3, inherited from his mother, and a c.1094T > A/p.I365N in exon 9, inherited from his father (Fig. 1a, b). Neither mutation was present in several databases. In the in silico analysis according to ANNOVAR, the prediction for the c.1094T > A (p.I365N) indicated a deleterious effect by SIFT, Polyphen-2, and MutationTaster. Fig. 1d–g shows the pathological findings of tissues. We present the pathological findings in the skin, brain, and kidney of our patient (Fig. 1d–g). However, no microscopic histopathological alternations caused by inflammation or infectious findings were observed in the gastrointestinal regions and lungs, instead of the clinical findings.

**Fig. 1:**
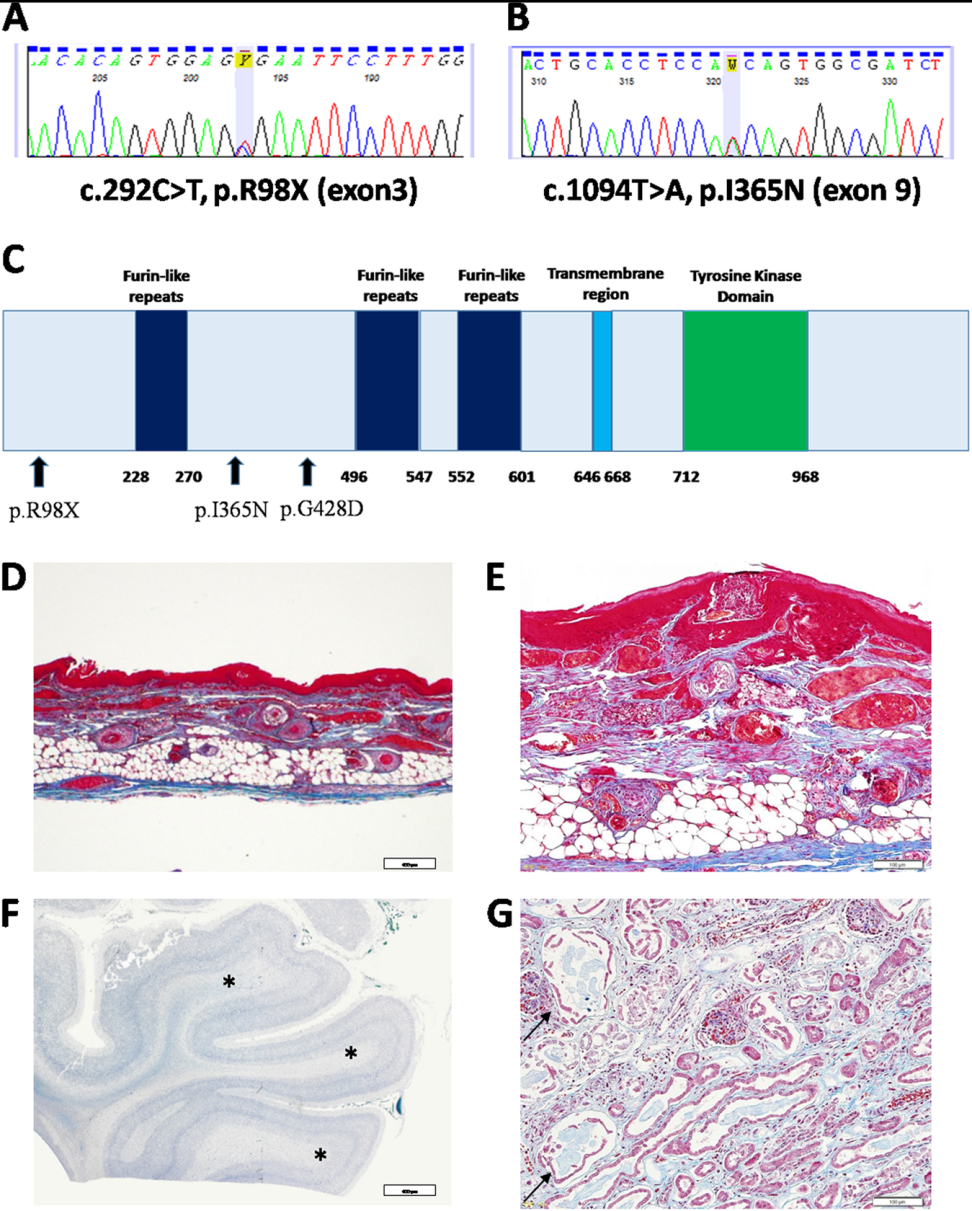
**(a**, **b)** Heterozygous mutations in *EGFR* detected in our patient. **a** Nonsense mutation, c.292C > T, p.R98X (exon 3). **b** Missense mutation, c.1094T > A, p.I365N (exon 9). **c** The schema of the location of domains and mutations in *EGFR*. **d–g** Histopathology of our patient: hematoxylin and eosin staining of brain tissue, Masson’s staining of dermal and kidney tissues. (**d**), **e** Skin tissue. The patient showed multiple subcorneal pustules on the skin. Decreasing dermal density and number of sweat glands. Hair follicles, eccrine glands, and sebaceous glands were reduced. **f** Brain. Hypomyelination of white matter (asterisks). **g** Kidney tissue. Multiple dilation of renal tubules (arrows) and expansion of the interstitium

Campbell et al.^4^ reported a male infant from a consanguineous family who possessed a homozygous missense mutation located in another extracellular domain of *EGFR*. Ganetzky et al.^5^ reported additional consanguineous family members with affected siblings caused by c.1283 G > A (p.Gly428Asp). Although the somatic gain-of-function mutations in the tyrosine kinase domain typically observed in malignant tumors, such as lung cancers, are commonly found, the LoF mutation might be present in ED patients; c.1283 G > A (p.Gly428Asp) was already demonstrated to be an LoF mutation located in the extracellular domain of *EGFR*, which might alter its structure, leading to potential dysfunction^4^. c.292 C > T / p.R98X was considered to be an LoF mutation because it is a nonsense mutation (Fig. 1c). Because we have not conducted a functional study of c.1094 T > A / p.I365N, it was unknown whether this mutation was an LoF mutation. The clinical courses of these patients were variable but were characterized by life-threatening infection or gastrointestinal futures. Cutaneous and digestive symptoms were common in the previous cases and in our patient (Table 1). Our patient and patient 2 from Granetzky et al.^5^ were severe cases and died at approximately three months of age. Although the progression of patient 3 was not mentioned, her condition appeared to be as severe as patient 2. Patient 1 was not as severely affected and was able to survive over two years of age. These facts indicate that there are currently no genotype–phenotype correlations. We attribute the difference in pulmonary condition not to mutation type but to our patient’s presence in the neonatal care unit, where he was unlikely to be exposed to bacteria that might cause respiratory infections. *EGFR* knockout mice show some clinical and pathological similarities with our patient^6–9^. Although EGFR-null mice show no pathological findings in any organs at birth, they subsequently develop them in the skin, lungs, and digestive organs. They are all exposed directly to foreign material, particularly pathogens, and thus have a specific immune system with a rapid cell-cycle frequency^10^. Taken together with the clinical findings, EGFR appears to be important for the development of organs that have a tissue-specific immune system after birth. Although our patient had no neurological symptoms, pathological findings showed hypomyelination of white matter. No evidence indicates that EGFR is involved in human brain development. The pathogenic findings of our patient thus give new insight into the function of EGFR in human brain development.

**Table 1:** Phenotypic characteristics of patients with biallelic EGFR mutations

	Campbell et al.	Ganetzky et al.		Present Case
	Patient 1	Patient 2	Patient 3	
Polyhydramnios	+	−	+	−
Premature birth (weeks)	34	34	33	32
IUGR	+	+	+	+
Birth weight (g)	1560	N.A	1195	1089
Mutation	p.G428D / p.G428D	p.G428D / p.G428D	p.G428D / p.G428D	p.R98X / p.I365N
Alopecia	+	+	+	+
Aged facial appearance	−	+	+	−
Pseudohydrocephalus	−	+	+	−
Skin desquamation	+	+	+	−
Ichthyosis	+	+	+	−
Acquired skin inflammation	+	N.A.	N.A.	+
Absent subcutaneous fat	−	+	+	−
Trichomegaly	+	−	−	−
Nephromegaly	+	+	+	−
Intestinal perforation	−	+	+	−
Recurrent vomiting/diarrhea	+	+	+	+
Recurrent infections	+	−	−	+
Respiratory difficulties	+	−	+	−
Others	−	−	−	Ventricular septal defect

*N.A* not available

In summary, we present the first ED patient with biallelic compound heterozygous mutations of EGFR. We propose that patients with biallelic mutations of EGFR can be diagnosed with ED, an autosomal recessive disease with a poor prognosis.

## Data Availability

The relevant data from this Data Report are hosted at the Human Genome Variation Database at 10.6084/m9.figshare.hgv.2306, 10.6084/m9.figshare.hgv.2309.
